# The Survival Advantage of Females at Premenopausal Age Is Race Dependent in Colorectal Cancer

**DOI:** 10.1155/2020/7434783

**Published:** 2020-12-30

**Authors:** Hui Sheng, Xiaoli Wei, Qunxi Chen, Kewei Huang, Runkun Han, Yijun Liu, Wen Liu, Minjie Mao

**Affiliations:** ^1^Department of Experimental Research, Sun Yat-sen University Cancer Center, State Key Laboratory of Oncology in South China, Collaborative Innovation Center for Cancer Medicine, Guangzhou 510060, China; ^2^Department of Medical Oncology, Sun Yat-sen University Cancer Center, State Key Laboratory of Oncology in South China, Collaborative Innovation Center for Cancer Medicine, Guangzhou 510060, China; ^3^Department of Pathology, Sun Yat-sen University Cancer Center, State Key Laboratory of Oncology in South China, Collaborative Innovation Center for Cancer Medicine, Guangzhou 510060, China; ^4^Department of Clinical Laboratory, Sun Yat-sen University Cancer Center, State Key Laboratory of Oncology in South China, Collaborative Innovation Center for Cancer Medicine, Guangzhou 510060, China

## Abstract

**Background:**

A female prognostic advantage in younger individuals has been demonstrated in various cancers. Several large-scale analyses based on different racial backgrounds have reported inconsistent results in colorectal cancer. The aim of the present study was to evaluate the prognostic value of sex and age in patients with colorectal cancer of different ethnic groups.

**Methods:**

We identified 71,812 eligible patients from the Surveillance, Epidemiology and End Results database. According to age at diagnosis, the patients were categorized into premenopausal age (≤45 yrs), menopausal age (46–54 yrs), and postmenopausal age (≥55 yrs) subgroups for further analysis.

**Results:**

Multivariate analysis identified the female survival advantage to be significant in the premenopausal age subgroup (*P* = 0.002, HR (95% CI): 0.73 (0.60–0.89)), diminished in the menopausal age subgroup (*P* = 0.09), and absent in the postmenopausal age subgroup (*P* = 0.96). Furthermore, the female survival advantage at premenopausal age was significant only in white patients (*P* = 0.001, HR (95% CI): 0.68 (0.54–0.87)) and not in either American Indian/Alaska Native or Asian or Pacific Islander patients. There was a trend of better survival of females in black patients (*P* = 0.07).

**Conclusions:**

Sex was a major prognostic factor in colorectal cancer patients, especially premenopausal women, and the difference was also associated with race.

## 1. Introduction

Colorectal cancer (CRC) is the most common malignancy in the digestive system worldwide. Globally, the estimated number of new cases was 1,096,601 in 2018, ranking fourth among all malignancies. Although the incidence and mortality of CRC has been declining in recent years, it is still the fourth leading cause of cancer-specific deaths worldwide [1]. The situation is even worse in China, where both the incidence and mortality of CRC are steadily increasing [2, 3]. Major treatment strategies include surgery, chemotherapy, and radiotherapy, the last of which is more common in rectal cancer [4–6]. Targeted therapies are restricted to late-stage cases, and only a few drugs have been approved for clinical application [7–9]. The identification of prognostic factors is important not only for survival prediction and patient management but also sometimes for developing new therapeutics.

The sex differences in cancer prognosis have been discussed for a long time. Previous studies have suggested that there is a survival advantage in female patients compared with male patients only or most notably at a younger age in several cancers, including melanoma [10, 11], nasopharyngeal carcinoma [12], hepatocellular carcinoma [13], metastatic oesophageal squamous cell carcinoma [14], and papillary thyroid cancer [15].

In CRC, there have been numerous studies investigating the role of sex in prognosis. Four studies based on large populations deserve more attention [16–19]. Unfortunately, the conclusions are inconsistent. These four studies are based on different geographic populations: Japan (*n* = 82,402), Taiwan (*n* = 62,060), Germany (*n* = 164,996), and the USA (*n* = 52,882). The studies based on German and American populations concluded that the survival advantage of females was only significant or more remarkable in younger populations [18, 19]. However, the studies based on Japanese and Taiwanese populations concluded that there were no survival differences between female and male patients under 50 years old [16, 17]. The different death rates among various races may be related to socioeconomic status, age, sex, tumour stage at diagnosis, and colorectal cancer screening programmes [20, 21]. However, after controlling for these variable characteristics, prognostic differences remained among the races [22]. The reasons for the ethnic differences may be genetic [23] or related to biological susceptibility [24] or microsatellite instability [25]. Thus, we conducted a large-scale analysis based on the Surveillance, Epidemiology, and End Results (SEER) Program CRC database to examine the prognostic value of sex and age in patients with colorectal cancer of different ethnic groups.

## 2. Materials and Methods

### 2.1. Ethics Statement

This study was deemed exempt from institutional review board approval by the Sun Yat-sen University Cancer Center, and informed consent was waived. This study was conducted in accordance with the ethical standards of the World Medical Association Declaration of Helsinki.

### 2.2. SEER Database

The SEER database includes cancer incidence and prevalence information tabulated by age, sex, race/ethnicity, year of diagnosis, and geographic region. It is publicly available. In this analysis, we used the latest version based on the November 2014 submission, “Incidence-SEER 18 Regs Research Data + Hurricane Katrina Impacted Louisiana Cases, Nov 2014 Sub (1973-2012 varying).”

### 2.3. Study Population

The cases were selected according to the third edition of the International Classification of Diseases for Oncology (ICD-O-3) codes. Colorectal (C18.0, C18.2–C18.7, and C19.9 for colon cancer and C20.9 for rectal cancer) adenocarcinoma (8140–8147, 8210–8211, 8220–8221, 8255, 8260–8263, 8480–8481, 8490, and 8574) was eligible for this study. Only cases with records of 7^th^ TNM stage from 2010 to 2012 were included. In addition, cases without follow-up records and with other tumours as the primary tumours were excluded.

### 2.4. Statistical Analysis

We used SPSS for Windows V.13.0 (SPSS Inc., Chicago, IL, USA) for all the analyses in this study. The sex differences in clinicopathologic characteristics were compared with the chi-square test (age, tumour location, and marital status) or Kruskal-Wallis *H* test (grade and TNM stage). In survival analyses, the end point was CRC-specific overall survival (OS), calculated as the time interval between CRC diagnosis and CRC-specific death or censored at death from other causes or the last record of follow-up. Univariate and multivariate Cox regression analyses were performed for the prognostic differences of sex and used to estimate hazard ratios (HRs) and 95% confidence intervals (95% CIs). Survival curves were plotted with the Kaplan-Meier method and compared using the log-rank test. A two-tailed *P* value < 0.05 was considered statistically significant.

## 3. Results

### 3.1. Basic Characteristics of the Study Population

This study identified 71,812 CRC patients from the SEER database. The basic characteristics of the study population are shown in the Supplementary Table 1. Male (52.1%, *n* = 37,448) patients were slightly more frequent than female (47.9%, *n* = 34,364) patients. According to the age distribution, 7.2% (*n* = 5,164) of patients were at premenopausal age (≤45 yrs) and 15.6% (*n* = 11,202) were at menopausal age (46–54 yrs), while most of the patients (77.2%, *n* = 55,446) were at postmenopausal age (≥55 yrs). Colon cancer (79.7%, *n* = 57,240) was more frequent than rectal cancer (20.3%, *n* = 14,572). There were 6,102 (8.5%) patients without available information for the tumour grade. Most of the tumours were moderately differentiated (72.0%, *n* = 47,287). Only 9.4% (*n* = 6,146) and 18.7% (*n* = 12,277) were well differentiated and poorly differentiated or undifferentiated, respectively. The frequencies of patients at stages 0, I, II, III, and IV were 3.4% (*n* = 2,475), 24.6% (*n* = 17,664), 27.2% (*n* = 19,546), 28.8% (*n* = 20,680), and 15.9% (*n* = 11,447), respectively. Regarding race, except for 611 (0.9%) patients without available information, most of the remaining patients were white (78.7%, *n* = 56,005), and the frequencies of the other three races were 0.7% (*n* = 520) for American Indian/Alaska Native, 8.7% (*n* = 6,214) for Asian or Pacific Islander, and 11.9% (*n* = 8,462) for Black. Regarding insurance and marital status, most patients were insured (83.8%, *n* = 58,753) and married (56.2%, *n* = 37,848).

### 3.2. Sex Differences of Clinicopathologic Characteristics

The median age in this study population was 66 years old. There were 53.0% patients at an older age (>66 yrs) in females, which was significantly higher than the 43.3% of male patients that were in this age group (*P* < 0.001). Rectal cancer was more frequent in males (23.3%) than in females (17.0%) (*P* < 0.001). The tumours of female patients were significantly more poorly differentiated than the tumours of male patients (*P* < 0.001). In addition, fewer of the female patients were married (46.4% vs. 65.2%), and more of the female patients were widowed (26.3% vs. 6.8%) than the male patients (*P* < 0.001). There were no significant differences in the sex distribution of the TNM stage (*P* = 0.98). Detailed information is shown in Table 1.

We further repeatedly conducted comparisons stratified by age (premenopausal age (≤45 yrs)/menopausal age (46–54 yrs)/postmenopausal age (≥55 yrs)). A significantly higher frequency of rectal cancer in females remained consistent among the three age subgroups (all *P* < 0.001). However, more female patients were married in the premenopausal age subgroup, and the rate subsequently declined in the menopausal and postmenopausal age subgroups, along with a corresponding increase in the rate of widowed patients among female patients. The distributions of marital status in the menopausal and postmenopausal age subgroups were consistent with those of the general study population (both *P* < 0.001). Interestingly, regarding tumour grade, female patients had significantly better tumour differentiation (*P* = 0.01) in the premenopausal age subgroup; however, this difference disappeared in the menopausal age subgroup (*P* = 0.6) and was even reversed in the postmenopausal age subgroup (*P* < 0.001). Detailed information is shown in Table 2.

### 3.3. Sex Difference in Colorectal Cancer-Specific Overall Survival Stratified by Age

**Figure 1:**
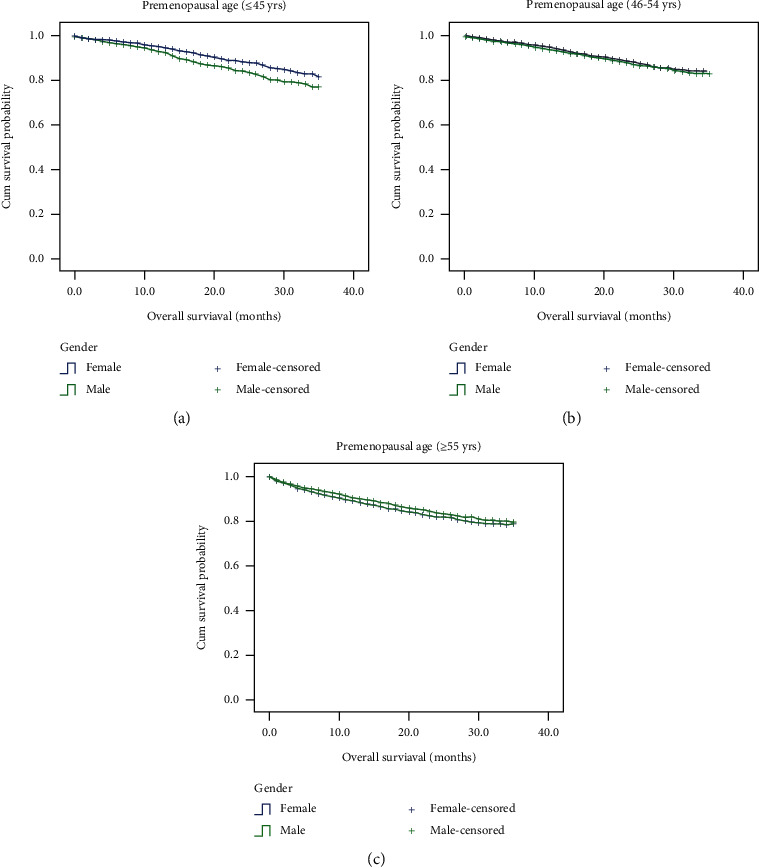
Sex differences in colorectal cancer-specific overall survival in colorectal cancer patients stratified by age. Survival was estimated by the Kaplan-Meier method and compared with the log-rank test, and a survival advantage in female patients was found in the premenopausal age subgroup (*P* < 0.001) that diminished in the menopausal age subgroup (*P* = 0.09) and was reversed in the postmenopausal age subgroup (*P* < 0.001).

Female patients had a significantly higher CRC-specific OS compared with male patients in the premenopausal age subgroup (*P* < 0.001, HR (95% CI): 0.70 (0.59–0.84)). This survival advantage of female patients diminished in the menopausal age subgroup (*P* = 0.09, HR (95% CI): 0.89 (0.79–1.02)) and reversed in the postmenopausal age subgroup (*P* < 0.001, HR (95% CI): 1.12 (1.07–1.17)) (Figure 1). Then, the prognostic differences of sex were adjusted by confounding factors, including tumour location (colon/rectum), grade (well differentiated/moderately differentiated/poorly differentiated or undifferentiated), TNM stage (0/I/II/III/IV, AJCC 7^th^), race (American Indian/Alaska Native/Asian or Pacific Islander/Black/White), insurance status (insured/others), marital status (married/widowed/others), and surgery for primary tumours (no/yes). The survival advantage of female patients remained independently significant in the premenopausal age subgroup (*P* = 0.002, HR (95% CI): 0.73 (0.60–0.89)), and there was still a trend of better survival for females in the menopausal age subgroup (*P* = 0.09, HR (95% CI): 0.88 (0.77–1.02)). However, there was no sex difference in CRC-specific OS in the postmenopausal age subgroup (*P* = 0.96, HR (95% CI): 0.96 (0.91–1.01)) after adjusting for confounding factors (Table 3).

### 3.4. Survival Advantage of Females at Premenopausal Age Stratified by Race

**Figure 2:**
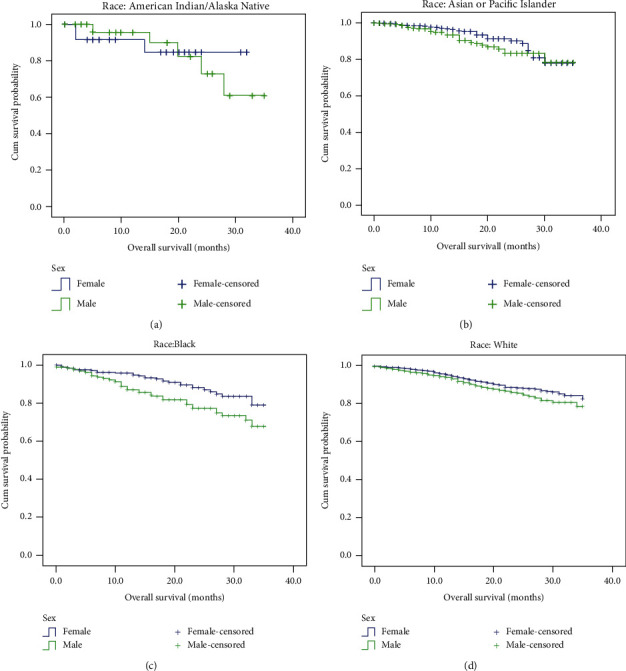
Sex differences in colorectal cancer-specific overall survival in colorectal cancer patients at premenopausal age stratified by race. Survival was estimated by the Kaplan-Meier method and compared with the log-rank test. The survival advantage of females only existed in black (*P* = 0.003) and white (*P* = 0.01) patients and not American Indian/Alaska Native (*P* = 0.94) or Asian or Pacific Islander (*P* = 0.36) patients.

Since we proved the survival advantage of females at the premenopausal age and we previously speculated that it was race dependent, we then analysed the sex differences in CRC-specific OS for patients at the premenopausal age among various races, as shown in Table 4. Univariate analyses indicated that the survival advantage of females only existed in black (*P* = 0.003, HR (95% CI): 0.52 (0.33–0.80)) and white (*P* = 0.01, HR (95% CI): 0.74 (0.60–0.92)) patients. There were no survival differences between males and females in American Indian/Alaska Native (*P* = 0.94, HR (95% CI): 0.94 (0.22–4.02)) or Asian or Pacific Islander (*P* = 0.36, HR (95% CI): 0.76 (0.43–1.37)) patients.  Figure 2 shows the sex differences in CRC prognosis at premenopausal age stratified by race. After adjusting for confounding factors, including tumour location, grade, TNM stage, insurance status, marital status, and surgery for primary tumours, a significant survival advantage of females was found in white patients (*P* = 0.001, HR (95% CI): 0.68 (0.54–0.87)), and there was a trend of better survival for females in black patients (*P* = 0.07, HR (95% CI): 0.61 (0.36–1.05)). However, no differences were found in either American Indian/Alaska Native (*P* = 0.99, HR (95% CI): 1.00 (0.16–6.25)) or Asian or Pacific Islander (*P* = 0.96, HR (95% CI): 1.01 (0.54–1.92)) patients.

## 4. Discussion

In this study, with a large CRC sample size from the SEER database, we verified the survival advantage of females in patients at premenopausal age and proved that this survival difference was race dependent.

The most convincing mechanism for the survival advantage of female patients with cancer was the protective value of female hormones. One powerful piece of evidence was the consistent female advantage of prognosis in younger patients in various cancers [10–15]. There has also been abundant evidence for the protective role of female hormones, mainly oestrogen, from cancer incidence, including CRC [26–29]. In a prospective study, oestrogen replacement therapy significantly reduced CRC risk with an RR of 0.81 (95% CI: 0.63–1.03) for users of 1 year or less, and the effect was more obvious for users of 11 years or more (RR and 95% CI: 0.54 (0.39–0.76)) [30]. Although the role of oestrogen in carcinogenesis, especially for breast cancer, has been well documented [31], it seemed to exert an inverse protective effect in CRC. The mechanism is complex and not fully understood. The oestrogen receptor beta has been demonstrated to play an important role. It was inversely associated with tumour stage and grade [32, 33] and might be a target for CRC prevention [34]. This could also be an explanation for the survival advantage of females at the premenopausal age identified in our study. In accordance with this hypothesis, we found females to be associated with better tumour differentiation at the premenopausal age but with worse tumour differentiation at the postmenopausal age.

In addition to the role of female hormones, there were also some other differences between male and female patients that could result in the survival advantage of females. Lifestyle behaviour differences were presumed to explain sex differences in prognosis; however, a study in nasopharyngeal carcinoma refuted this speculation [12]. Some genetic differences, such as the VEGF-2587 CC genotype [35], a change in the oestrogen receptor alpha/beta ratio [36], polymorphic variants of oestrogen receptor beta [37], and the ROCK1 rs35996865 G variant allele [38], were also identified as possible mechanisms. In addition, a different reaction to radiation was observed between female and male patients [39]. A longer half-life of cisplatin in females has also been demonstrated. All these factors would support better survival in female cancer patients.

The age of female patients was significantly older than that of male patients in our study. This result was in accordance with previous reports. Ferlitsch et al. found that the prevalence of advanced adenomas was comparable between males aged 45–49 and females aged 55–59 [40]. Brenner et al. found that females mainly reached equivalent levels of CRC incidence when 4–8 years older than males [41]. This delayed incidence pattern might also be a result of the protective value of female hormones. The difference in age distribution could also partially explain the higher rate of widowed patients in females found in our study. However, a higher rate of widowed patients was identified across all age subgroups, which could possibly indicate a different role of marital status in CRC susceptibility.

It is clear that there are some differences in cancer incidence and survival among people of different racial backgrounds [42]. A survival advantage of Asian patients compared with white patients has been found in several cancers, including breast cancer [43], gastric cancer [44], non-small-cell lung cancer [45], and CRC [46]. Disparities of social, economic, and lifestyle behaviour factors might partially be responsible; however, with the implementation of genomic approaches, differences in genetic and epigenomic profiles among various races have been revealed, and the influence of race on cancer biology has been demonstrated [42].

The major limitation of this study was that we did not include lifestyle behaviour factors in the analysis because they were not available in the SEER database. Additionally, without data on menopausal status and hormonal analysis, we could only roughly categorize patients into three menstrual subgroups according to age. Furthermore, the occurrence and development of tumours is a very complex process. Due to the heterogeneity of tumours, every independent study may have bias. Since the Asian sample size in the SEER database is insufficient, the result is limited. The differences found in the present study among races may only reflect the impact of some biological, social, and behavioural differences among races. More studies are needed to reveal intrinsic impact factors. The major strength of our study was the relatively large sample size based on SEER registry data, and the biggest highlight of our study was that we demonstrated for the first time that the survival advantage of females at premenopausal age is race dependent.

## 5. Conclusions

Our study was the first to discover the race dependence of female survival advantage at the premenopausal age, which existed only in white patients and possibly in black patients but was not present in American Indian/Alaska Native or Asian or Pacific Islander patients. This was consistent with previous large-scale reports. How race-related and sex-related genetic or other factors interact with each other in CRC needs further investigation.

## Figures and Tables

**Table 1:** Sex differences in clinicopathologic characteristics in colorectal cancer.

Characteristic	Male *N* (%)	Female *N* (%)	*P* value
Age (yrs, median: 66)			<0.001^∗^
≤66	21,223 (56.7)	16,151 (47.0)	
>66	16,225 (43.3)	18,213 (53.0)	
Tumour location			<0.001^∗^
Colon	28,707 (76.7)	28,533 (83.0)	
Rectum	8,741 (23.3)	5,831 (17.0)	
Grade			<0.001^∗^
Well differentiated	3,247 (9.6)	2,899 (9.1)	
Moderately differentiated	24,892 (73.3)	22,395 (70.5)	
Poorly differentiated or undifferentiated	5,814 (17.1)	6,463 (20.4)	
TNM stage			0.98
0	1,369 (3.7)	1,106 (3.2)	
I	9,286 (24.8)	8,378 (24.4)	
II	9,977 (26.6)	9,569 (27.8)	
III	10,702 (28.6)	9,978 (29.0)	
IV	6,114 (16.3)	5,333 (15.5)	
Marital status			<0.001^∗^
Married	22,925 (65.2)	14,923 (46.4)	
Widowed	2,398 (6.8)	8,465 (26.3)	
Others	9,851 (28.0)	8,804 (27.3)	

^∗^Significant *P* value. Abbreviation: TNM: tumour-node-metastasis.

**Table 2:** Sex differences in clinicopathologic characteristics in colorectal cancer stratified by age.

Characteristic	Premenopausal age (≤45 yrs)			Menopausal age (46–54 yrs)			Postmenopausal age (≥55 yrs)		
	Male *N* (%)	Female *N* (%)	*P* value	Male *N* (%)	Female *N* (%)	*P* value	Male *N* (%)	Female *N* (%)	*P* value
Tumour location			<0.001^∗^			<0.001^∗^			<0.001^∗^
Colon	19,00 (70.4)	1,861 (75.5)		4,446 (71.4)	3,802 (76.5)		22,361 (78.4)	22,870 (84.9)	
Rectum	799 (29.6)	604 (24.5)		1,783 (28.6)	1,171 (23.5)		6,159 (21.6)	4,056 (15.1)	
Grade			0.01^∗^			0.6			<0.001^∗^
Well differentiated	180 (7.3)	155 (6.8)		549 (9.9)	475 (10.5)		2,518 (9.7)	2,269 (9.1)	
Moderately differentiated	1,668 (68.1)	1,664 (72.9)		4,113 (74.1)	3,338 (73.5)		19,111 (73.6)	17,393 (69.8)	
Poorly differentiated or undifferentiated	602 (24.6)	463 (20.3)		892 (16.1)	729 (16.1)		4,320 (16.6)	5,271 (21.1)	
TNM stage			0.18			0.45			0.54
0	77 (2.9)	68 (2.8)		274 (4.4)	209 (4.2)		1,018 (3.6)	829 (3.1)	
I	400 (14.8)	441 (17.9)		1,513 (24.3)	1,189 (23.9)		7,373 (25.9)	6.748 (25.1)	
II	606 (22.5)	523 (21.2)		1,356 (21.8)	1,064 (21.4)		8,015 (28.1)	7.982 (29.6)	
III	1,009 (37.4)	876 (35.5)		1,920 (30.8)	1,581 (31.8)		7,773 (27.3)	7,521 (27.9)	
IV	607 (22.5)	557 (22.6)		1,166 (18.7)	930 (18.7)		4,341 (15.2)	3,846 (14.3)	
Marital status			0.001^∗^			<0.001^∗^			<0.001^∗^
Married	1,453 (56.4)	1,398 (59.8)		3,680 (63.3)	2,786 (60.0)		17,792 (66.4)	10,739 (42.6)	
Widowed	7 (0.3)	19 (0.8)		50 (0.9)	126 (2.7)		2,341 (8.7)	8,320 (33.0)	
Others	1,115 (43.3)	919 (39.3)		2,079 (35.8)	1,730 (37.3)		6,657 (24.8)	6,155 (24.4)	

^∗^Significant *P* value. Abbreviation: TNM: tumour-node-metastasis.

**Table 3:** Univariate and multivariate analyses for the prognostic value of sex stratified by age.

Age group	Univariate		Multivariate^§^	
	*P* value	HR (95% CI)	*P* value	HR (95% CI)
Premenopausal age (≤45 yrs)	<0.001^∗^	0.70 (0.59–0.84)	0.002^∗^	0.73 (0.60–0.89)
Menopausal age (46–54 yrs)	0.09	0.89 (0.79–1.02)	0.09	0.88 (0.77–1.02)
Postmenopausal age (≥55 yrs)	<0.001^∗^	1.12 (1.07–1.17)	0.13	0.96 (0.91–1.01)

^∗^Significant *P* value. Abbreviation: TNM: tumour-node-metastasis. ^§^Sex differences of prognosis were adjusted by tumour location (colon/rectum), grade (well differentiated/moderately differentiated/poorly differentiated or undifferentiated), TNM stage (0/I/II/III/IV), race (American Indian/Alaska Native/Asian or Pacific Islander/Black/White), insurance status (insured/others), marital status (married/widowed/others), and surgery for primary tumours (no/yes) in multivariate analysis.

**Table 4:** Univariate and multivariate analyses for the prognostic value of sex at premenopausal age stratified by race.

Race (*N* (%))	Univariate		Multivariate§	
	*P* value^∗^	HR (95% CI)	*P* value	HR (95% CI)
American Indian/Alaska Native (56 (1.1))	0.94	0.94 (0.22–4.02)	0.99	1.00 (0.16–6.25)
Asian or Pacific Islander (496 (9.7))	0.36	0.76 (0.43–1.37)	0.96	1.01 (0.54–1.92)
Black (696 (13.6))	0.003^∗^	0.52 (0.33–0.80)	0.07	0.61 (0.36–1.05)
White (3,865 (75.6))	0.01^∗^	0.74 (0.60–0.92)	0.001^∗^	0.68 (0.54–0.87)

^∗^Significant *P* value. Abbreviation: TNM: tumour-node-metastasis. ^§^Sex differences of prognosis were adjusted by tumour location (colon/rectum), grade (well differentiated/moderately differentiated/poorly differentiated or undifferentiated), TNM stage (0/I/II/III/IV), insurance status (insured/others), marital status (married/widowed/others) and surgery for primary tumours (no/yes) in multivariate analysis.

## Data Availability

The data used to support the findings of this study are available from the corresponding author upon request.

## Supplementary Materials

Supplementary Materials Supplementary Table 1: basic characteristics of the study population.
