# Supplementary material for: The Survival Advantage of Females at Premenopausal Age Is Race Dependent in Colorectal Cancer
Source: Biomed Res Int. 2020 Dec 30;2020:7434783. doi: 10.1155/2020/7434783 (PMC7787739; doi:10.1155/2020/7434783)
Supplement: Supplementary Materials — Supplementary Table 1: basic characteristics of the study population. [file 7434783.f1.docx]

**Supplementary Table 1.** Basic characteristics of the study population.

| Characteristics | Number (%) |
| --- | --- |
| Sex |  |
| Male | 37,448 (52.1) |
| Female | 34,364 (47.9) |
| Age (yrs) |  |
| Premenopausal age (≤ 45) | 5,164 (7.2) |
| Menopausal age (46 – 54) | 11,202 (15.6) |
| Postmenopausal age (≥ 55) | 55,446 (77.2) |
| Tumor location |  |
| Colon | 57,240 (79.7) |
| Rectum | 14,572 (20.3) |
| Grade |  |
| Well differentiated | 6,146 (9.4) |
| Moderately differentiated | 47,287 (72.0) |
| Poorly differentiated or undifferentiated | 12,277 (18.7) |
| TNM stage |  |
| 0 | 2,475 (3.4) |
| Ⅰ | 17,664 (24.6) |
| Ⅱ | 19,546 (27.2) |
| Ⅲ | 20,680 (28.8) |
| Ⅳ | 11,447 (15.9) |
| Race |  |
| American Indian/Alaska Native | 520 (0.7) |
| Asian or Pacific Islander | 6,214 (8.7) |
| Black | 8,462 (11.9) |
| White | 56,005 (78.7) |
| Insurance status |  |
| Insured | 58,753 (83.8) |
| Others | 11,344 (16.2) |
| Marital status |  |
| Married | 37,848 (56.2) |
| Widowed | 10,863 (16.1) |
| Others | 18,655 (27.7) |

Abbreviation: TNM, tumor-node-metastasis.
